# Construction of yeast microbial consortia for petroleum hydrocarbons degradation

**DOI:** 10.3389/fbioe.2024.1408361

**Published:** 2024-05-09

**Authors:** Minzhen Wang, Mengyu Zhou, Hengchang Li, Zhibei Cao, Mingzhu Ding, Yingjin Yuan

**Affiliations:** ^1^ Frontiers Science Center for Synthetic Biology and Key Laboratory of Systems Bioengineering (Ministry of Education), School of Chemical Engineering and Technology, Tianjin University, Tianjin, China; ^2^ Frontiers Research Institute for Synthetic Biology, Tianjin University, Tianjin, China

**Keywords:** biodegradation, n-hexadecane, alkane hydroxylases, microbial consortia, glutathione

## Abstract

Microbial degradation of petroleum hydrocarbons plays a vital role in mitigating petroleum contamination and heavy oil extraction. In this study, a *Saccharomyces cerevisiae* capable of degrading hexadecane has been successfully engineered, achieving a maximum degradation rate of up to 20.42%. However, the degradation ability of this strain decreased under various pressure conditions such as high temperature, high osmotic pressure, and acidity conditions. Therefore, a *S*. *cerevisiae* with high tolerance to these conditions has been constructed. And then, we constructed an “anti-stress hydrocarbon-degrading” consortium comprising engineered yeast strain SAH03, which degrades hexadecane, and glutathione synthetic yeast YGSH10, which provides stress resistance. This consortium was able to restore the degradation ability of SAH03 under various pressure conditions, particularly exhibiting a significant increase in degradation rate from 5.04% to 17.04% under high osmotic pressure. This study offers a novel approach for improving microbial degradation of petroleum hydrocarbons.

## 1 Introduction

Petroleum hydrocarbon biodegradation plays a crucial role in addressing environmental pollution caused by oil spills during petroleum production, storage, transportation, and refining processes (Cao et al., 2022). It has emerged as a burgeoning field of research, with promising applications in microbial enhanced oil recovery (MEOR) techniques. Microorganisms contribute to MEOR through two main mechanisms (Wu et al., 2022). Firstly, they degrade long-chain alkanes into shorter chain compounds, reducing the carbon content and enhancing the flowability of heavy crude oil, thereby increasing oil recovery rates. Secondly, microorganisms produce organic solvents such as acids and alcohols that react with carbonate minerals in reservoir pores, expanding the pore space and reducing oil viscosity, ultimately improving oil recovery rates. However, extreme environmental conditions, such as high temperature, high osmotic pressure, and acidity, can adversely affect microbial growth and degradation capabilities (Liu et al., 2015). Therefore, the stress resistance of microorganisms is crucial for efficient petroleum hydrocarbon degradation.

Most of the identified microorganisms capable of degrading petroleum hydrocarbons belong to non-model microorganisms, such as *Mycobacterium sp.* (Wu et al., 2020), *Rhodococcus sp*. (Gibu et al., 2019), and *Pseudomonas sp*. (Chettri et al., 2021). To gain a deeper understanding of the degradation mechanism, scientists have conducted research on the degradation pathways of petroleum hydrocarbons and enzymes in strains for petroleum hydrocarbons degradation. Among them, four degradation pathways have been identified for alkanes (>C_5_): terminal oxidation pathways (Watkinson and Morgan, 1990), subterminal oxidation pathways (Forney and Markovetz, 1970), diterminal oxidation pathways (Kester and Foster, 1963), and the Finnerty pathway (Ji et al., 2013). Regarding alkane-degrading enzymes, significant progress has been made in the study of alkane hydroxylases. Integral-membrane alkane hydroxylases (AlkB, AlkM) (Ratajczak and Hillen, 1998; Bihari et al., 2011), cytochrome P450 alkane hydroxylases (CYP153C1) (Craft et al., 2003; Zhou et al., 2011), flavoprotein alkane hydroxylases (LaoA, AlmA) (Feng et al., 2006; Throne-Holst et al., 2007) have been identified and studied.

With the clarification of degradation pathways, it becomes feasible to utilize model organisms to construct engineered strains that can be precisely controlled exogenously in model microorganisms. In research, *Trametes trogii* Lac (*Ttlcc1*) was engineered into *S*. *cerevisiae* strain CEN.PK2–1°C to obtain high hydrocarbon resistance and degradation capability (Asemoloye and Marchisio, 2022). However, engineered strains constructed exogenously face challenges in adapting to external complex environments. Therefore, the establishment of microbial consortia has emerged as a crucial approach to address this issue. In hydrocarbon-degrading microbial consortia system, different strains collaborate in various ways. For example, alkane-degrading strains and surfactant-secreting strains, alkane-degrading strains and nitrogen-providing strains, and strains with their own preferences for alkanes and aromatic hydrocarbons, cooperate with each other in the medium with petroleum hydrocarbons as the only carbon source (Piehler et al., 1999; Chen et al., 2014; Liu et al., 2021). With the development of synthetic biology, designing a consortium system that efficiently degrades petroleum hydrocarbons while adapting to harsh environments has become a research hotspot.

This study aims to explore methods for engineered yeast to degrade long-chain alkanes, using n-hexadecane as a representative substrate. *S. cerevisiae*, a model eukaryote with a complete organelle and heterologous protein expression system, was chosen as the microbial chassis. Based on this, an engineered yeast strain capable of efficiently degrading petroleum hydrocarbon pollutants and an engineered yeast strain capable of producing glutathione (γ-glutamylcysteinylglycine, GSH) was constructed by introducing exogenous genes. Furthermore, a microbial consortium system was constructed to restore the degradation ability of the engineered yeast under harsh conditions.

## 2 Materials and methods

### 2.1 Strains, media and cultivation conditions

The transformation was initiated using *S. cerevisiae* strain BY4741 (Jia et al., 2018). *Escherichia coli* Trans1-T1 (Trans Gene Biotech, Beijing, China) was employed for plasmid maintenance and amplification. All yeast strains utilized in this study are listed in Table 1. Yeast strains were selectively inoculated in synthetic complete medium (SC) composed of 6.7 g/L yeast nitrogen base, appropriate amino acid dropout mixture, and approximately 20 g/L glucose. Yeast strains were preserved by being stored in 30% glycerol at −80°C. Unless stated otherwise, chemicals were obtained from Sigma-Aldrich (St. Louis, MO, United States).

**TABLE 1:** Strains used in this study.

Strains	Description	Source
*S*. *cerevisiae* BY4741	—	Library
SAH01	BY4741 harboring pRS416-*alkM*	This study
SAH02	BY4741 harboring pRS426-*alkM*	This study
SAH03	BY4741 harboring pRS416-*alkM*-linker-*rubA*	This study
SAH04	BY4741 harboring pRS426-*alkM*-linker-*rubA*	This study
SAH05	BY4741 harboring pRS416-*alkM*-linker-*rubA*-linker-*rubB*	This study
SAH06	BY4741 harboring pRS426-*alkM*-linker-*rubA*-linker-*rubB*	This study
YGSH01	BY4741 harboring pRS416-*sod1*	This study
YGSH02	BY4741 harboring pRS416-*ttc0189*	This study
YGSH03	BY4741 harboring pRS416-*gsh1*	This study
YGSH04	BY4741 harboring pRS416-*gsh2*	This study
YGSH05	BY4741 harboring pRS416-*gsh61512*	This study
YGSH06	BY4741 harboring pRS416-*gpx.km*	This study
YGSH07	BY4741 harboring pRS416-*gpx.wm*	This study
YGSH08	BY4741 harboring pRS416-*grx2*	This study
YGSH09	BY4741 harboring pRS416-*grx35095*	This study
YGSH10	BY4741 harboring pRS416-*grl1*	This study
YGSH11	BY4741 harboring pRS416-*pos5*	This study
YGSH12	BY4741 harboring pRS416-*nk60672*	This study

### 2.2 DNA manipulation

All bacterial genes, including *alkM* (alkane hydroxylase gene) and its coenzyme genes *rubA* and *rubB* were codon-optimized and obtained from Kingsley company. Promoter and terminator sequences fragments were amplified from the genome of *S. cerevisiae*. The recombinant plasmids used in this study were based on modified pRS vector series.

In brief, gene expression units for each enzyme were created in the pRS vector, surrounded by different potent promoters and terminators (Zhou et al., 2021). Unique restriction sites (BamHI/HindIII) were introduced on either side of each gene expression cassette. To enhance recombination efficiency, homologous fragments were utilized to construct plasmids for fusion protein expression. For strain construction, the plasmids were transformed into *E. coli* and engineered *E. coli* strains were established through colony PCR verification. Plasmids were isolated from *E. coli*, and yeast strains were then genetically modified using the LiAc/SS carrier DNA/PEG technique (Agatep et al., 1998). The transformed cells were subsequently selected on appropriate synthetic dropout agar plates (without uracil or leucine) for transformation into *S. cerevisiae* BY4741.

### 2.3 Shake flask cultivation

An isolated yeast strain was cultured in 5 mL of suitable SC medium with 20 g/L glucose for a minimum of 12 h. After that, 50 mL of the corresponding synthetic yeast medium was added to a 250 mL flask, starting with an OD_600_ of 0.2. The fermentation medium was enriched with 10 g/L n-hexadecane. The strain is cultivated in a shaking flask at a temperature of 30°C, pH value of 6, shaking speed of 220 rpm, and a flask volume ratio of 5:1. To assess the hexadecane degradation rate, samples were taken from each flask after 96 h of cultivation. The inoculation ratio of strains is 1:1, with an initial total OD_600_ of 0.2. The microbial consortium was cultivated under single factor-controlled conditions at 35°C, 5% NaCl, and pH = 5.

### 2.4 GC/MS analysis of the hydrocarbon degradation

The n-hexadecane content in each supernatant was extracted using an equal volume (1:1 v/v) of ethyl acetate. Afterwards, 1 mL of the polar phase was analyzed using a Thermo Scientific DSQII single quadrupole gas chromatography mass spectrometry (GC/MS) system with an HP-5MS column (operating at a pulsed split ratio of 1:1 and a split flow rate of 1 mL/min). The oven temperature was first set and maintained at 40°C for 3 min, followed by a gradual temperature increase at a rate of 6°C/min up to 300°C, with a final 2-min hold at that temperature. The quantification of n-hexadecane is achieved by comparing the peak areas of the test sample with those of the standard sample. The amount of n-hexadecane was adjusted by considering its mass spectrometer response factors, which were determined by analyzing dilution series of commercial standards.

## 3 Results

### 3.1 The construction of *S. cerevisiae* for degradation

**FIGURE 1:**
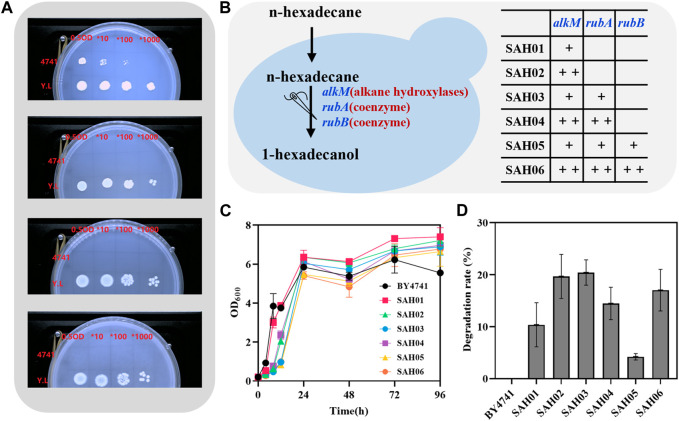
Construction of *S. cerevisiae* degrading n-hexadecane. **(A)** from top to bottom, the culture-medium of plates is respectively SC, SC-Glucose, SC-Glucose+ Tween 40 and SC-Glucose + Tween 40+ n-Hexadecane; **(B)** construction diagram of engineered *S. cerevisiae*; **(C)** the growth curve of engineered *S. cerevisiae*; **(D)** the 96 h degradation rate of engineered *S. cerevisiae* to n-hexadecane.

We constructed an engineered strain capable of degrading hexadecane based on the *S. cerevisiae* BY4741. To assess the hexadecane degradation ability of wild strain (*S. cerevisiae* BY4741), BY4741 and *Yarrowia lipolytica* were cultured in SC medium until mid-log phase, followed by washing and starvation treatment for 48 h, and subsequently subjected to spot plate experiments. It is known that *Y*. *lipolytica* can degrade hexadecane. By comparing the growth of BY4741 and *Y. lipolytica*, we validated the ability of wild strain to degrade hexadecane. The results showed that wild strain could not utilize hexadecane as the sole carbon source for growth (Figure 1A).

Next, we attempted to introduce the alkane hydroxylase AlkM into wild strain using the single-copy plasmid pRS416, resulting in the strain SAH01 (Figure 1B). To observe the hexadecane degradation ability of this strain, we measured the OD_600_ values of the strain within 96 h and the hexadecane degradation rate after 96 h. The results revealed that SAH01 exhibited better growth in media containing hexadecane and glucose as carbon sources compared to wild strain (Figure 1C), with a hexadecane degradation rate of 10.37% for SAH01 (Figure 1D).

To further enhance the hexadecane degradation ability of the engineered strain, the exogenous gene *alkm* was subjected to multicopy optimization to generate the strain SAH02. The OD_600_ values of SAH02 approached those of SAH01 after 24 h and this trend continued until 48 h. By 72 h, the OD_600_ value of SAH02 was slightly lower than that of SAH01, but by 96 h, both strains had OD_600_ values exceeding 7.0. SAH02 exhibited growth lag during the logarithmic growth phase (Figure 1C). Degradation rate analysis indicated a significant improvement in the engineered yeast strain SAH02 with multicopy optimization compared to SAH01, reaching 19.68% (Figure 1D). This suggests that the introduction of multiple copies of the alkane hydroxylase gene, while imposing a higher growth burden on the strain, enhances its hexadecane degradation ability. This outcome is analogous to the synthesis of vitamin C in *S. cerevisiae* (Zhou et al., 2021).

The genes *rubA* and *rubB* encode for the coenzymes rubredoxin and rubredoxin reductase of the alkane hydroxylase AlkM, respectively (Ratajczak and Hillen, 1998), which play crucial roles in electron transfer during the enzymatic process. To enhance the catalytic effect of alkane hydroxylase and improve degradation efficiency in engineered yeast, the alkane hydroxylase was fused with its coenzymes. To ensure that the structure of the alkane hydroxylase and its coenzymes would not negatively impact each other due to proximity, a linker sequence was inserted between the two genes while constructing the plasmid containing the target genes. Subsequently, the *alkM* gene fused with different coenzyme genes was separately ligated to single-copy and multicopy vectors, obtaining four strains: SAH03, SAH04, SAH05, and SAH06. Results from growth conditions and degradation rate determination experiments indicated that among the strains using the single-copy vector, three strains exhibited similar trends in OD_600_ values, with the order of SAH01 > SAH03 > SAH05. The degradation rates of these strains were found to be SAH03 > SAH01 > SAH05. Notably, the strain SAH03, which was introduced with a single coenzyme, achieved the highest degradation rate of 20.48%, which was 1.97 times higher than the strain without coenzyme introduction. In contrast, among the strains using a multi-copy vector, a distinct difference in growth was observed after 24 h, with OD_600_ values being SAH02 > SAH04 > SAH06. Moreover, the introduction of coenzymes resulted in a decrease in degradation rates of the strains. Specifically, compared to the control strain SAH02, the degradation rates of SAH04 and SAH06, which introduced one and two coenzymes respectively, decreased by 5.21% and 2.04%. Interestingly, we found that the growth of strains weakened with the introduction of coenzymes, but the corresponding degradation rates did not show a complete positive correlation with the growth conditions.

### 3.2 The construction of stress-resistant *S. cerevisiae*

**FIGURE 2:**
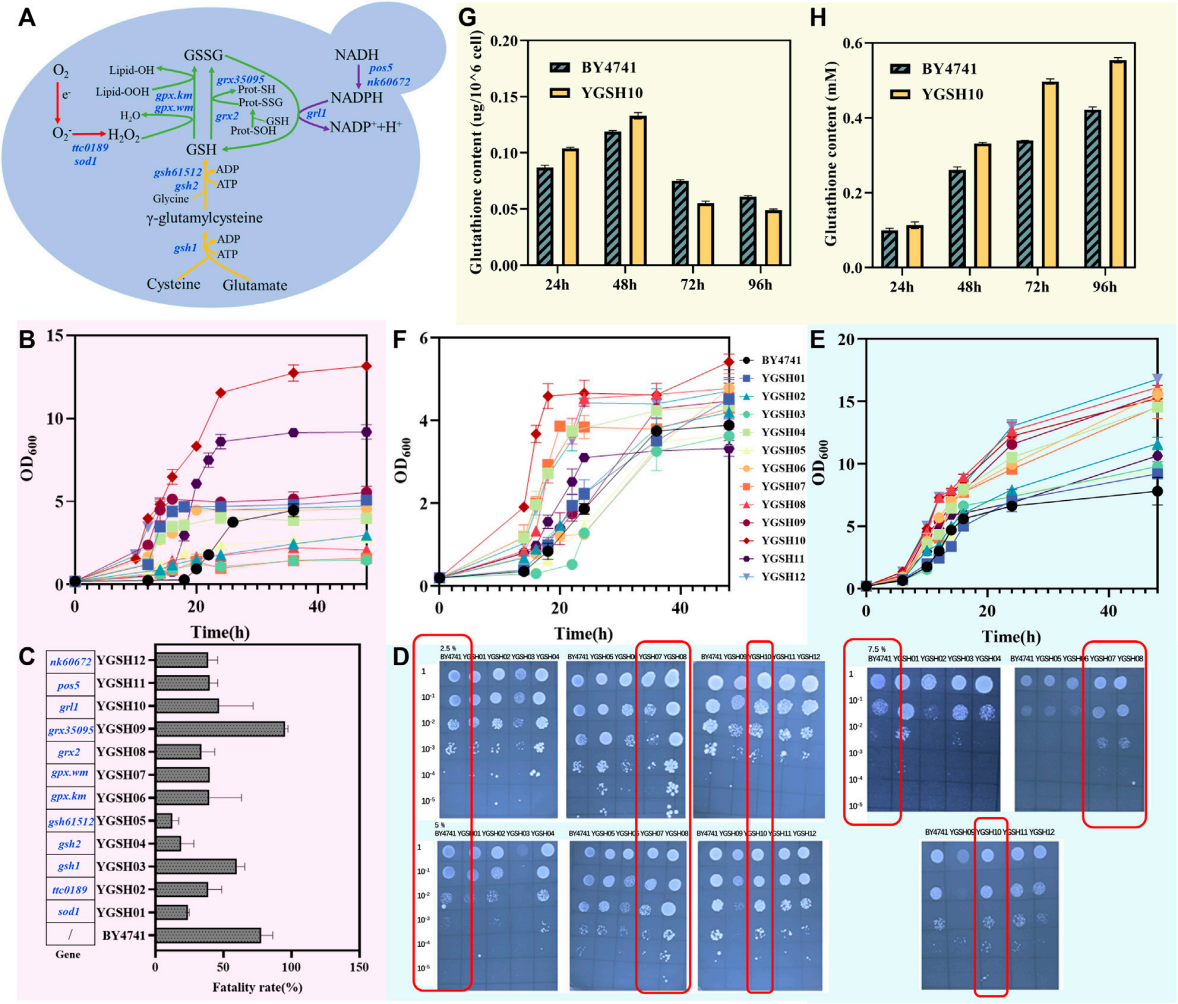
The construction of stress-resistant *S. cerevisiae*. **(A)** schematic diagram of the GSH synthesis and recycling process:red represents the SOD module, green represents the GSH oxidation module, yellow represents the GSH synthesis module, and purple represents the GSH reduction module; **(B)** graph depicting the variation of cell concentration over time for the yeast strain under heat stress at 40°C; **(C)** mortality rate of the yeast strain following heat shock at 55°C; **(D)** growth of twelve YGSH on SC-ura plate containing 2.5%, 5% and 7.5% NaCl; **(E)** graph depicting the variation of cell concentration over time for the yeast strain under osmotic stress at 5% NaCl; **(F)** graph depicting the variation of cell concentration over time for the yeast strain under inorganic acid stress (HCl, pH = 3.0); **(G)** intracellular GSH production of YGSH10; **(H)** extracellular GSH production of YGSH10.

The GSH synthesis and cycling process were divided into four modules: SOD module, GSH synthesis module, GSH oxidation module, and GSH reduction module (Pastore et al., 2003). The principal reaction in the SOD module involves the clearance of superoxide anions, catalyzed by the SOD enzyme (Dubois-Randé et al., 1994). The GSH synthesis module is composed of two reactions, each catalyzed by different GSH synthesizing enzymes. The GSH oxidation module primarily encompasses the oxidation of GSH to oxidized glutathione (GSSG), catalyzed by glutathione peroxidase (GPx) and glutaredoxin (Grx) (Grant et al., 1996). This oxidation process simultaneously reduces H_2_O_2_, phospholipid hydroperoxides, and protects thermosensitive thiol proteins. The GSH reduction module bifurcates, first catalyzing the conversion of NADH to NADPH through NADH kinase to generate reducing power, and then reducing GSSG to GSH via glutathione oxidoreductase. *Kluyveromyces marxianus* exhibits elevated thermotolerance in yeasts (Cui et al., 2023), *Whereas mellicola* (Sun et al., 2019) is an acid-resistant marine bacterium, and *Thermus thermophilus* HB27 (Gallo et al., 2021) is an extremophilic thermophile. These microbes demonstrate substantial tolerance under various conditions. Consequently, the twelve genes selected for this study are derived from the three microorganisms. After undergoing codon optimization provided by a commissioned company, these genes were introduced into the *S. cerevisiae* BY4741 (Figure 2A).

To validate the strain tolerance during growth at 40°C, yeast strains YGSH01-YGSH12, along with BY4741, were inoculated into YPD liquid medium at a final OD_600_ of 0.2 and cultured under 40°C at 220 rpm for 48 h. As shown in Figure 2B, strain YGSH10 exhibited notably superior heat resistance compared to other strains, characterized by a brief lag phase, prolonged log phase, high cell concentration, swift recovery from heat-induced damage, and sustained, enhanced cell vitality at 40°C. Although strain YGSH11 exhibited slower recovery of cell activity from high temperatures, its log phase was shorter than that of YGSH10, indicating higher cell vitality under heat, and its final OD_600_ was second only to YGSH10. Both YGSH10 and YGSH11 express the GSH reduction module, suggesting a pivotal role of GSH reduction module in yeast’s defense against heat damage. Additionally, strains YGSH12, YGSH09, YGSH01, YGSH06, and YGSH04 (order from fast to slow) could recover cell vitality at a relatively quick pace under high-temperature stress, reaching cell concentrations comparable to wild strain (*S. cerevisiae* BY4741). Compared to BY4741, the remaining strains YGSH03, YGSH07, YGSH08, YGSH02, and YGSH05 (order from strong to weak) showed noticeably enhanced sensitivity to heat. Moreover, no significant secondary growth phenomena were observed for all twelve yeast strains and BY4741 at 40°C.

To further validate the heat tolerance of strains at 55°C, secondary seed cultures were obtained and diluted to an OD_600_ of 1 using sterile water. For the experimental group, the cultures underwent heat shock at 55°C in a metal bath for 15 min while control groups remained untreated. Three biological replicates per group were conducted. Yeast cell counts under both conditions were obtained through gradient dilution plating, and the cell mortality induced by 55°C heat shock was calculated. As shown in Figure 2C, except for strain YGSH09, the remaining eleven strains exhibited lower cell mortality rates compared to wild strain under 55°C heat shock, with more cells surviving. The poorer heat tolerance of YGSH09 may be attributed to the gene *grx35095* sourced from *W. mellicola*, which has lower homology with yeast. Among YGSH01-YGSH12, YGSH05 exhibited the strongest heat tolerance at 55°C, followed by YGSH04 and YGSH01, while the mortality rate of the remaining strains was around 50%. Divided per module, strains from the SOD and GSH reduction modules displayed higher cell survival rates under 55°C heat stress compared to others. It is inferred that high-temperature heat shock may necessitate increased intracellular O^2-^ clearance speed and maintaining GSH in a reduced state to protect mitochondria from oxidative damage, thereby sustaining normal life activities.

To ascertain the NaCl concentration that induces osmotic stress and preliminarily understand the strains’ osmotic tolerance, the study cultivated YGSH01-YGSH12, along with wild strain expressing pRS416, on SC-ura solid plates with varying NaCl concentrations. Figure 2D displays the outcomes: the twelve yeast strains seemingly exhibited no significant variance in their defense against osmotic stress caused by NaCl. However, the growth conditions of three strains, YGSH07, YGSH08, and YGSH10, were superior to BY4741 (pRS416) on solid plates containing 5% and 7.5% NaCl. For a more accurate characterization of strain tolerance, YGSH01-YGSH12, along with BY4741, were inoculated in YPD liquid medium containing 5% NaCl and cultured for 48 h. The results, as shown in Figure 2E, categorized the twelve yeast strains into two groups based on salt tolerance: YGSH12, YGSH08, YGSH09, YGSH06, YGSH10, YGSH07, and YGSH04 (order from strong to weak) exhibited enhanced salt tolerance compared to the wild-type strain; whereas YGSH02, YGSH11, YGSH03, YGSH05, and YGSH01 (order from strong to weak) showed salt tolerance slightly higher than or equivalent to BY4741. Furthermore, compared to H_2_O_2_ and thermal stress, 5% NaCl had a milder impact on yeast growth, characterized by a short lag phase and higher final OD_600_. The influence of 5% NaCl on yeast growth predominantly occurred during the log phase, slowing the growth rate. Moreover, according to module classification, the three strains from the GSH reduction module and two from the GSH oxidation module exhibited higher osmotic tolerance. This suggests that upregulation of exogenous gene expression related to GSH cycling positively affected yeast growth under salt stress induced by NaCl.

To validate the strains’ tolerance to inorganic acid, yeast strains YGSH01-YGSH12, along with BY4741, were inoculated to a final OD_600_ of 0.2 and cultivated in SC-ura liquid medium (acidified with HCl) at pH 3.0 for 48 h. YGSH10 uniquely exhibited a secondary growth phase among the twelve strains, recovering cellular vitality rapidly from the inorganic acid stress (Figure 2F). Following YGSH10, strains YGSH08, YGSH12, YGSH07, and YGSH04 demonstrated resilience under inorganic acid stress, with a final OD_600_ around 4.0. The remaining strains displayed a sensitivity to inorganic acid similar to that of BY4741. Categorized by module, strains from the GSH reduction module exhibited superior inorganic acid tolerance. This study infers that maintaining a reduced state of GSH in yeast cells under inorganic acid stress is crucial for cellular protection, especially in preventing mitochondrial oxidative damage and sustaining normal intracellular life processes (Outten and Culotta, 2004; Outten et al., 2005).

The intracellular and extracellular GSH levels were measured in the most tolerant strain YGSH10 among the 12 engineered strains. The results showed that YGSH10 could secrete more glutathione compared to the wild strain BY4741. At 24 h and 48 h, the intracellular GSH content in YGSH10 was higher than that in BY4741. However, at 72 h and 96 h, the intracellular GSH content in YGSH10 was lower than that in BY4741 (Figure 2G). Meanwhile, the extracellular GSH content of YGSH10 was higher than that of the yeast BY4741. The highest increase in GSH content for YGSH10 was observed at 24–48 h, reaching 0.218 mM. From 48 h to 72 h, the increase in GSH content for YGSH10 was 2.13 times higher than that of BY4741 (Figure 2H). These findings indicate that between the 48–72 h period, YGSH10 secretes a significant quantity of intracellular GSH into the extracellular environment, resulting in a reduction of intracellular GSH and an elevation of extracellular GSH.

### 3.3 The construction of microbial consortia system

#### 3.3.1 Effect of exogenous GSH on engineered yeast under different conditions

Microorganisms are subjected to a variety of extreme environments due to the complexity and variability of the external environment, such as high temperature, high osmotic pressure, and acidity (Halliwell, 2006). Many of these conditions are unfavorable for yeast growth and often result in oxidative damage. Glutathione plays a crucial role in oxidative stress in *S. cerevisiae*. In this study, we selected the engineered yeast strain SAH03 and investigated the restorative role of GSH under high temperature (35°C), high osmotic pressure (5% NaCl), and acidic conditions (pH = 5).

**FIGURE 3:**
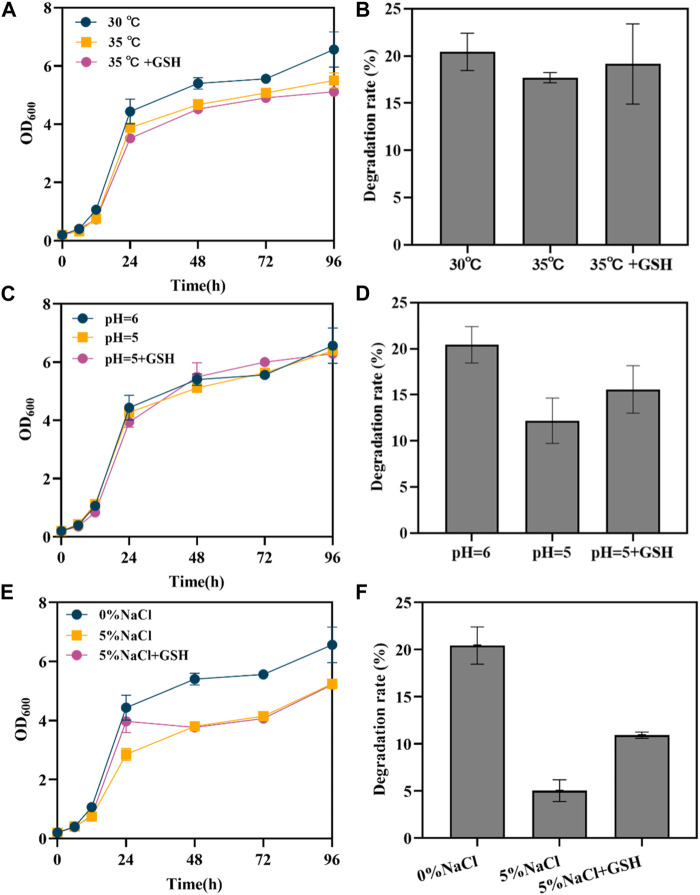
Effect of exogenous GSH on engineered yeast under different conditions. **(A)** the growth curve of SAH03 at high temperature; **(B)** the 96 h degradation rate of SAH03 to n-hexadecane at high temperature; **(C)** the growth curve of SAH03 at acidic condition; **(D)** the 96 h degradation rate of SAH03 to n-hexadecane at acidic condition; **(E)** the growth curve of SAH03 at high osmotic pressure; **(F)** the 96 h degradation rate of SAH03 to n-hexadecane at high osmotic pressure.

Yeast generally grows best at temperatures between 28°C–30°C. At 35°C, the growth of the engineered yeast strain was inhibited, evidenced by an elongated lag phase and a slower entry into the logarithmic growth phase. Strains with exogenous GSH added to the culture medium showed slightly higher OD_600_ values than those without GSH during the 0–12 h period, suggesting a restorative effect of GSH on strain growth (Figure 3A). Compared to the degradation rate of 20.42% for strains grown at 30°C, the degradation rate under high temperature conditions fell to 17.69%. For engineered yeast strains with exogenous GSH added to the culture medium, the degradation rate recovered to 19.14%, which is 1.08 times that of strains without GSH (Figure 3B). The results indicate that high-temperature conditions (35°C) reduced the degradation rate of the strains, and the addition of GSH had little effect on the recovery of the strains’ degradation capabilities.

The normal pH for yeast SC medium is 6. Growth of yeast was not significantly inhibited under acidic conditions (pH = 5). During the 0–24 h period, the growth curves of strains grown in acidic conditions and normal conditions nearly overlapped. After 24 h, the OD_600_ values of strains grown under acidic conditions gradually fell below those grown under normal condition (Figure 3C). It is speculated that this is due to acid production during strain growth. In the early stage, the amount of acid produced was insufficient to impact strain growth. However, after producing a certain amount of acid in the later stage, the pH of the culture medium further decreased, reaching a threshold that influenced strain growth, and the inhibitory effects of acidity started to manifest. Acidic conditions inhibited the degradation of hexadecane, lowering the degradation rate of engineered strains from 20.42% to 12.19%. However, the addition of exogenous 286 GSH increased the degradation rate to 15.59%, which is 1.28 times that of strains grown without GSH (Figure 3D). The results indicate that exogenous GSH has a restorative effect on the reduction of degradation rate caused by acidic conditions in strains.

Under 5% NaCl osmotic pressure, the growth of engineered yeast is inhibited, but exogenous GSH exhibits a strong restorative effect on its growth. After 24 h, the growth advantage of strains grown in culture medium with exogenous GSH was no longer apparent, and their OD_600_ values gradually equated to those of strains grown without GSH (Figure 3E). This is speculated to be due to the gradual oxidation of GSH, weakening its restorative effect. High osmotic pressure inhibited both strain growth and degradation ability, reducing the degradation efficiency from 20.42% to 5.04%. However, the degradation rate of engineered strains with exogenous GSH was 10.91%, 2.16 times that of strains without GSH (Figure 3F).

From the restorative effects of exogenous GSH on strain degradation rates under the three conditions, the ability of GSH to function differs under different conditions. Glutathione showed the best restorative effect under high osmotic pressure, followed by acidic conditions, with the poorest performance under high temperature conditions.

#### 3.3.2 Effects of YGSH10 on engineered yeast SAH03 under different conditions

According to the results in 3.3.1, exogenous GSH can help to restore the degradation rate of engineered degradable yeast under stress conditions. Therefore, an “anti-stress hydrocarbon-degrading” consortium system was established, comprised of SAH03 and YGSH10. The growth and degradation rates of this consortium were studied under normal conditions (30°C, 0% NaCl, pH = 6), high temperature (35°C), high osmotic pressure (5% NaCl), and acidic conditions (pH = 5).

**FIGURE 4:**
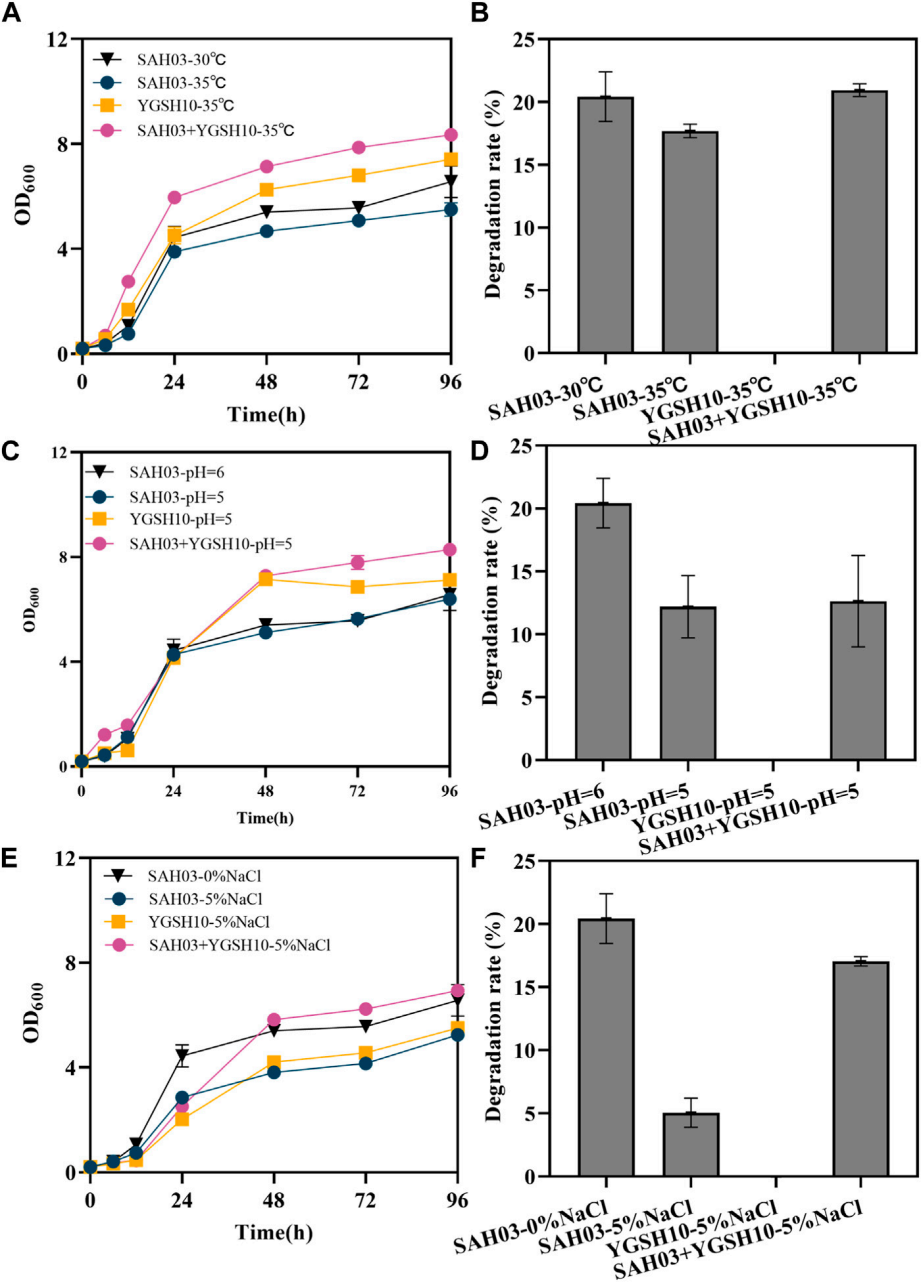
Effects of YGSH10 on SAH03 under different conditions. **(A)** the growth curve of SAH03, YGSH10 and consortium at high temperature; **(B)** the 96 h degradation rate of SAH03 to n-hexadecane at high temperature; **(C)** the growth curve of SAH03, YGSH10 and consortium at acidic condition; **(D)** the 96 h degradation rate of SAH03 to n-hexadecane at acidic condition; **(E)** the growth curve of SAH03, YGSH10 and consortium at high osmotic pressure; **(F)** the 96 h degradation rate of SAH03 to n-hexadecane at high osmotic pressure.

High temperature inhibited the growth of the yeast. The final OD_600_ values for SAH03 could reach 6.563 at 30°C, but under 35°C, it dropped to 5.502. In comparison, the growth of YGSH10 was better than that of SAH03 under high temperature, and the growth of the SAH03+YGSH10 system was best (Figure 4A). Under 35°C conditions, the degradation rate of SAH03 decreased by 2.43%, to 17.69%. However, the degradation rate of the microbial consortium could recover to 20.94%, 1.18 times that of SAH03 alone under the same conditions, fully restoring the degradation rate. Therefore, YGSH10 can aid in the recovery of SAH03’s degradation ability under high temperature conditions (Figure 4B).

Under acidic conditions, microbial consortium do not significantly enhance the degradation rate recovery of SAH03. The final OD_600_ values were ordered as follows: “SAH03+YGSH10-pH = 5” > “YGSH10-pH = 5” > “SAH03-pH = 6” > “SAH03-pH = 5” (Figure 4C). The degradation rate of SAH03 under acidic conditions is 12.19%, which decreased by 8.23%. Meanwhile, the degradation rate of the SAH03-YGSH10 system only reached 12.62%, only 0.43% higher than that of SAH03 alone (Figure 4D).

The addition of 5% NaCl exogenously inhibited the growth of the yeast, characterized by a decreased OD_600_ value (Figure 4E). At 96 h, the consortium reached the highest OD_600_ value of 6.934, which was 1.32 times that of SAH03 and 1.26 times that of YGSH10. Thus, the consortium helped recover the growth of SAH03. The degradation rate of SAH03 decreased by 18.08%, to 5.04%. However, the degradation rate of the microbial consortium could recover to 17.04%, 3.38 times that of SAH03 under the same conditions. Under conditions of 5% NaCl addition, the consortium could recover 83.47% of the degradation rate of the engineered strain SAH03. The results indicate that YGSH10 had a strong recovery effect on the engineered degradation yeast under high osmotic pressure (Figure 4F).

The recovery effects of the consortium on the degradation ability of SAH03 under these three different conditions exhibit diverse impacts. Among them, the consortium’s recovery effect is most pronounced under high osmotic pressure, followed by high temperature, and least effective under acidic conditions.

## 4 Discussion

With the advancement of synthetic biology and metabolic engineering techniques, microbial remediation technology is increasingly regarded as one of the most effective methods to address petroleum contamination. *S. cerevisiae*, a model organism in the fungi kingdom with complete organelles and heterologous protein expression system, has shown broad prospects for development aided by the progression in synthetic biology. Scientists have developed a synthetic chromosome recombination and modification by loxP-mediated evolution in *S. cerevisiae* that holds promise for further exploration (Jia et al., 2018). In the present study, we chose this yeast as the microbial chassis to introduce genes encoding alkane degradation enzymes/cofactors, creating an engineered yeast strain capable of efficiently degrading petroleum hydrocarbon pollutants.

Petroleum hydrocarbon-degrading yeast may encounter harsh environmental conditions in nature such as high temperature, high osmotic pressure, and varied pH, which are unfavorable for microbial growth and petroleum hydrocarbon degradation capability. GSH with active cysteine residues serves as an efficient antioxidant in *S. cerevisiae*, protecting the cells from oxidative damage. Recent researches suggested that strengthening GSH synthesis through molecular biological techniques like Cre-loxP system can enhance the tolerance of yeast strains (Zhu et al., 2011). Qiu et al., 2015 increased the copy number of GSH synthesizing enzyme gene (GCSGS), resulting in a threefold increase in GSH accumulation, enabling the yeast to withstand high temperatures up to 40°C. Ask et al. improved the robustness of *S. cerevisiae* in simultaneous saccharification and fermentation processes of pretreated spruce by regulating intracellular GSH levels (Smirnova et al., 2005). Recently, Diederik et al. found that yeasts could collectively clean their environments by reducing harmful extracellular reactive oxygen species, thereby assisting each other and their progeny to replicate and survive under high temperatures (Laman Trip and Youk, 2020). These findings suggest a role for GSH in constructing resistant microbial consortia. Moreover, studies indicate that an enhancement in GSH can amplify the electron-transferring capability of NADH (Cortes-Rojo et al., 2020). This becomes especially pertinent when considering enzymes like AlkM, a membrane-integrated non-heme iron monooxygenase (Williams and Austin, 2022), which necessitates cofactors and NADH for electron transfer.

In the study, an “anti-stress hydrocarbon-degrading” consortium comprising SAH03, an engineered yeast strain capable of degrading hexadecane, and YGSH10, a GSH-synthesizing strain, was established. Under stress conditions, a discernible restoration of SAH03’s degradation capacity was observed, attributed to YGSH10’s excretion of excess GSH. This possibly mitigated the detrimental extracellular reactive oxygen species, purifying the milieu and aiding the replication and survival of degrading yeast SAH03 and its progeny under adverse conditions. Furthermore, GSH could potentially elevate the enzymatic activity of the exogenous alkane hydroxylase AlkM through NADH, consequently enhancing the strain’s degradation capacity. However, under the same pressure condition, the difference between the recovery effect of exogenous GSH on engineered yeast strain SAH03 and the recovery effect of microbial consortium on it indicates that the mechanism of the recovery of degradation ability of microbial consortium on engineered yeast strain SAH03 may be more complex, and further research is needed. An alkane degradation pathway in *S. cerevisiae* was successfully constructed via synthetic biology in the study, and effective petroleum hydrocarbon degradation was achieved. It suggests that *S. cerevisiae* has great potential in degrading petroleum hydrocarbon pollution. Further construction of degradation pathways may lead to even more efficient degradation.

## Data Availability

The raw data supporting the conclusion of this article will be made available by the authors, without undue reservation.
